# Innovating to Improve Individualized Training in Internal Medicine Residency: Inpatient Threads and Block 2.0

**DOI:** 10.1007/s11606-025-10016-8

**Published:** 2025-12-02

**Authors:** Bryn Boslett, Lekshmi Santhosh, David Chia, Sarah Goglin, Nia Boykin, Julia Toy, Rebecca A. Berman

**Affiliations:** University of California, San Francisco, San Francisco, CA USA

## Abstract

**Background:**

The ACGME Internal Medicine (IM) Program Requirements mandate 6 months of individualized training and 10 months of outpatient medicine over 3 years. Balancing these with service needs is challenging. At UCSF, we developed a multi-pronged approach to individualize training across clinical settings.

**Aim:**

To develop a curriculum that supports individualized education, longitudinal mentorship, and career development while meeting ACGME requirements.

**Setting:**

An IM residency spanning three hospitals at the University of California, San Francisco.

**Participants:**

All PGY2–PGY3 categorical IM residents in the 2024–2025 academic year.

**Program Description:**

Two complementary innovations were implemented: (1) Inpatient Threads, allowing residents to select inpatient tracks aligned with career goals (e.g., generalist, cardiology/critical care); and (2) Block 2.0, a redesigned outpatient/elective curriculum with longitudinal subspecialty clinics (LSCs), skill-based pathways, and scholarly time. Residents completed 2 LSCs annually and enrolled in a GME-wide Academic Pathway.

**Program Evaluation:**

The redesign was informed by needs assessments and survey data collected from 2014 to 2023. Early outcomes signal improved mentorship and record participation in the resident research symposium. Ongoing evaluation includes ACGME survey data, internal surveys, and focus groups.

**Discussion:**

This customizable curriculum offers a replicable model to individualize training while preserving the generalist foundation of IM residency.

## INTRODUCTION

The ACGME’s Internal Medicine (IM) Residency Program Requirements identify individualized learning and increased ambulatory exposure as key priorities for residency training. IM residents must complete at least 6 months of individualized educational experiences tailored to their future practice or to further competency development in foundational areas along with a marked increase in the ambulatory footprint to a minimum of 10 months.^1,2^ There is no national standard nor best practices for residency programs to achieve this level of individualization.^3^ As a result, programs must navigate the challenges of balancing this individualization imperative with other training requirements and service priorities.

**Figure 1:**
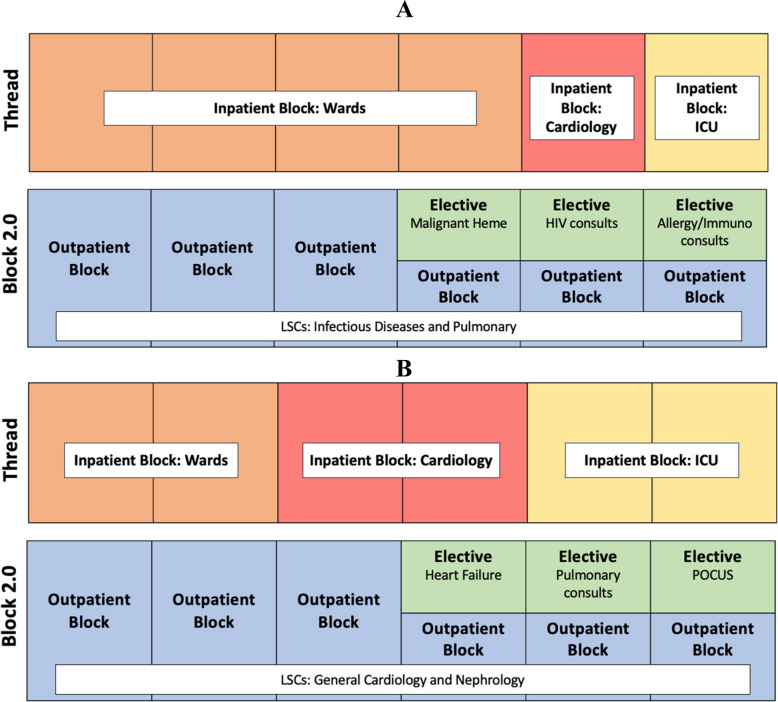
**Example academic year schedules.** **Residents follow a 4 + 4 schedule, alternating every 4 weeks between inpatient and outpatient/elective blocks. A) An infectious diseases (ID)-bound resident might select the General Categorical Thread for inpatient blocks, paired with outpatient LSCs in ID and pulmonary, and use electives for HIV, allergy/immunology, and oncology. B) A cardiology-bound resident may choose the Critical Care/Cardiology Thread, with cardiology and nephrology LSCs during outpatient blocks and electives in heart failure, pulmonary, and point-of-care ultrasound (POCUS).**

In parallel, a review of our residents’ self-assessments and program evaluations over the past 10 years highlighted the need for greater flexibility and enhanced experiences to better support their diverse learning needs and career goals, while complying with ACGME requirements. Here, we describe the creation, implementation, and evaluation of two major curricular interventions designed to personalize training in both inpatient and outpatient settings: **Inpatient Threads** and **Block 2.0** (Fig. 1).

**Inpatient Threads** allow residents to gain autonomy and individualization in their inpatient schedule. It introduces hospital-based residency tracks during inpatient time, allowing residents to pre-select a rotation pathway focused on preparing them for careers in general medicine, non-procedural subspecialties, or procedural subspecialties.

**Figure 2:**
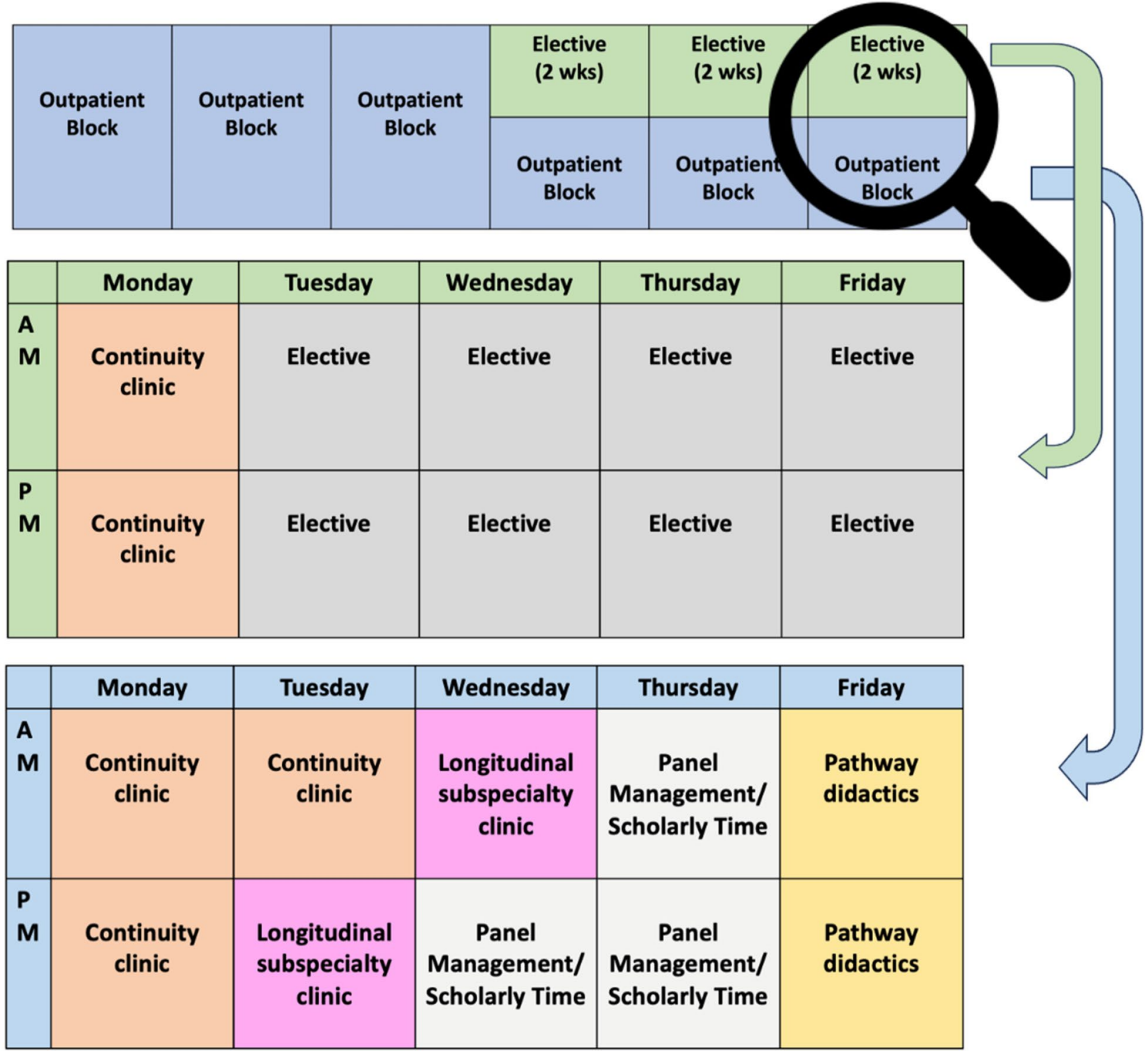
**Block 2.0 templates**. **Residents spend roughly half the academic year on “block time,” composed of elective blocks (6 weeks) and outpatient blocks (16 weeks). Elective blocks are immersive, with 1 day per week reserved for continuity clinic. Outpatient blocks are more varied and include continuity clinics, longitudinal subspecialty clinics (LSCs), Pathway didactics, and scholarly time.**

**Block 2.0** reimagines individualized learning blocks during outpatient time by incorporating longitudinal subspecialty clinics (LSCs), skill-based pathways, and scholarly projects, which are selected by PGY2 and PGY3 residents in collaboration with their mentors (Fig. 2). These curricular innovations, spanning six alternating months of outpatient and elective rotations per year, are tailored to residents’ career goals.

## SETTING AND PARTICIPANTS

The University of California, San Francisco (UCSF) Internal Medicine Residency Program consists of 46 categorical and 18 primary care residents per class. This academic year, we have a total of 185 residents. Training takes place across three core sites: UCSF Health, San Francisco General Hospital, and San Francisco Veterans Affairs Medical Center.

During the PGY-2 and PGY-3 years, residents follow a **4 + 4 model**, alternating every 4 weeks between inpatient and outpatient/elective experiences (Fig. 1). Inpatient rotations include general medicine wards, critical care, cardiology, night float, and jeopardy. Outpatient and elective experiences encompass continuity clinics, longitudinal subspecialty clinics, core and individualized didactics, and scholarly project time.

To individualize the curriculum while balancing ACGME requirements and clinical service needs, a **curricular redesign task force** was assembled. This group included key stakeholders—program leaders, educators, education staff, and residents. Over 4 months, the task force met weekly, incorporating resident input through town halls, surveys and program-wide communications.

## PROGRAM DESCRIPTION

Our previous curricular framework featured inpatient wards, critical care, and cardiology rotations evenly distributed among residents and a traditional elective model where residents completed subspecialty inpatient or outpatient consult rotations. Residents could request specific inpatient experiences if additional time was available, but the process was logistically cumbersome. Residents selected up to three subspecialty electives per year, representing the primary means of individualizing training. Feedback suggested this model fell short in supporting meaningful differentiation and career development. Manual scheduling without a rotation template increased administrative burden and led to inconsistent individualization.

In response, the redesign task force performed a needs assessment using 5 years of program-wide surveys of residents (response rates 31–68%) along with exit survey data. All graduating residents participate in required exit interviews with an Associate Program Director, with near 100% completion. Responses were de-identified and analyzed for themes in learner experience and curricular needs. Two major concerns emerged: (1) limited continuity with faculty mentors and (2) insufficient longitudinal exposure to chosen specialties. Beyond learner perspectives, the task force also considered features of the learning environment, including the availability of subspecialty clinics, preceptor capacity, and scheduling infrastructure. Together, these findings underscored the need for a curriculum that better supported longitudinal relationships, mentorship, and career-aligned training while remaining compliant with ACGME requirements and scheduling feasibility.

Recognizing that longitudinal learning enhances clinical growth and mentorship—an approach well supported by evidence in both undergraduate and graduate medical education ^4–9^—the task force sought to embed this principle intentionally within the residency experience. We therefore launched a comprehensive curricular redesign spanning both inpatient and outpatient environments as described below.

### Inpatient Threads: Structured Career-Aligned Tracks

The inpatient experience for all PGY2 and PGY3 residents was restructured into curricular tracks—Inpatient Threads—designed to align rotations with career goals while maintaining equitable scheduling. Each resident selects a predefined set of rotations tailored to their interests:

- **General Categorical Thread**—Designed for future generalists and non-procedural subspecialists (e.g., rheumatology, endocrinology) and includes 50% wards blocks, 25% critical care blocks, and 25% cardiology blocks across the PGY2 and PGY3 years. In addition, night float blocks were prioritized for wards.
- **Critical Care and Cardiology Thread**—Designed for future cardiologists, intensivists, and procedural subspecialists, and includes 33% wards blocks, 33% critical care blocks, and 33% cardiology blocks. In addition, night float blocks were prioritized for critical care and cardiology.

This structured approach provides residents with a “prix-fixe” menu of rotations that facilitates individualized training while improving balance and scheduling efficiency. Primary Care residents follow a thread similar to the General Categorical Thread, with an additional 2–4 weeks of ambulatory time per year. For clarity, this pathway is distinct but parallel to the categorical options. Of note, the number of residents selecting each respective pathway was similar in our first 2 years of implementation.

### Block 2.0: Individualized Outpatient and Elective Training

Recognizing that much of medicine occurs in the ambulatory setting, we redesigned our ambulatory teaching toward longitudinal subspecialty clinics (LSCs) along with our longitudinal primary care clinics. Each categorical PGY2 and PGY3 resident now completes 16 weeks of outpatient rotations and 6 weeks of electives annually, with a focus on immersive experiences tailored to career interests. The PGY1 schedule was minimally impacted by these changes as interns do not have elective time. As such, individualized scheduling is largely concentrated in PGY2 and PGY3 years.

Key components of Block 2.0 include:

- **Longitudinal subspecialty clinics (LSCs):**Residents pair with preceptors in their subspecialty of interest and see patients throughout their outpatient blocks. A single LSC has been an optional PGY2 experience since 2014, consistently receiving high marks from residents and faculty.^8^ In LSCs, residents gain broader exposure to ambulatory subspecialty diseases, more opportunities for career exploration, and stronger mentoring relationships with their subspecialist preceptors. Given their success, the program expanded to require two LSCs per year for both PGY2 and PGY3 residents. This model allows residents to obtain longitudinal ambulatory experience not only in their future subspecialty but in adjacent domains or growth areas identified on In-Training Exams.
- **Electives:** Two-week elective blocks, with 1 day per week protected for continuity clinic. Residents select three electives per year, which may be clinical or procedural, with one elective per year permitted in a non-clinical area (e.g., wellness, research).
- **Pathways:** UCSF Pathways have existed for over 20 years^10^ and are shown to support career development and academic engagement.^10–13^ Pathways provide longitudinal mentorship and a structured curriculum in areas such as bench science, clinical research, health systems innovation, global health, educational scholarship, health equity, and informatics. Previously optional, Pathways are now a required component of Block 2.0, with didactic time embedded into the schedule.
- **Scholarly time:** Dedicated time for Pathway mentorship and project completion. Residents are strongly encouraged to present their work at an annual resident research symposium.
- **Continuity clinic:** The existing continuity clinic structure was preserved to maintain primary care continuity, ensuring a predictable cadence for residents, patients, and preceptors.

Together, these curricular innovations create a structured yet flexible approach to individualized learning, providing longitudinal mentorship, greater ambulatory exposure, and career-aligned training while preserving core residency requirements.

## PROGRAM EVALUATION

The development of **Block 2.0** and **Inpatient Threads** was guided by structured needs assessments and prior program data. Because this innovation is in its inaugural year, formal outcome data are not yet available. We therefore present early outcomes and outline our planned evaluation strategy.

Prior data on longitudinal subspecialty clinics (LSCs) supported our outpatient curricular redesign. LSCs, introduced in 2014 for a subset of residents, were previously evaluated in a study of resident and faculty perceptions between 2014 and 2019. Among respondents, 87% (81/93) rated LSCs as very good or excellent in educational value, and 71% (66/93) reported that LSCs facilitated mentorship.^8^ Recent exit interviews from 2023 to 2024 echoed these findings, supporting the decision to expand LSCs and make them a required part of Block 2.0. Additional LSC-specific data are forthcoming to determine if expanded LSCs increase comfort in the practice of ambulatory medicine.

The requirement of Pathway participation and dedicated time for scholarly activity may be associated with an observed change in resident research productivity. This year’s annual resident research symposium saw a record-breaking number of submissions—61 abstracts with 54 unique submitting authors, up from an average of 37 per year (range 29–53) in the prior 5 years. We cannot yet establish causality; future data will be needed to determine whether this represents a sustained trend attributable to curricular change.

To formally assess the impact of these curricular innovations, future evaluation will include focus groups and formal end-of-year survey data to assess mentorship, satisfaction, and scholarly productivity. This data will help refine and optimize the program moving forward.

## DISCUSSION

Our Threads and Block 2.0 curricular redesign allows residents to create distinctive clinical and skills-based schedules that maximize training in their niche of choice and expand exposure to longitudinal approaches to care while fulfilling core requirements, clinical service needs, and reducing the burden of scheduling. During the initial year, PGY2 and PGY3 categorical residents were able to select an Inpatient Thread with a procedural or non-procedural emphasis and tailor their outpatient, elective, and didactic experiences toward a career path or specific skill set of interest. 100% of residents received their first or second choice of LSCs and electives, and all Thread requests have been fulfilled to date. We have also significantly increased deep ambulatory clinical experiences in subspecialty fields through the longitudinal subspecialty clinics (LSCs), which were well received by both residents and faculty in early pilots and were expanded in this new model.

The emphasis on longitudinal mentorship and subspecialty relationship-building is particularly impactful in an academic medical center like UCSF, where inpatient teaching is largely led by hospitalists, limiting subspecialty exposure during traditional ward rotations. Through this redesign, residents engage regularly with potential career mentors in both clinical and non-clinical settings and have greater access to protected time for research and scholarly pursuits. We have seen early indications that this investment is leading to increased scholarly output for our residents.

Our curricular intervention had several limitations. First, the expansion of LSCs from approximately 40 to over 180 experiences annually—two per categorical PGY2 and PGY3 resident—posed logistical challenges in identifying sufficient subspecialty faculty preceptors. While our large academic setting made this feasible, institutions with fewer resources may face constraints in implementation. Additionally, some residents (~ 5 annually) request to switch their LSCs or Threads during the academic year as career decisions change. This adds yet another layer of complexity with scheduling. We allow changes in Threads at the change of the academic year and have been able to accommodate LSC changes for the handful of residents with a change of heart career-wise. Second, the increase in protected didactic and scholarly time was sometimes misperceived as unstructured time. We addressed this through attendance tracking and reinforcement of professionalism expectations. This remains an area for ongoing monitoring. Third, the emphasis on individualized scheduling initially led some residents to overfocus on a single subspecialty area. In response, we instituted a limit of two experiences per subspecialty per year (i.e., one LSC and one elective) to ensure training breadth and maintain generalist foundations. Finally, we acknowledge the absence of pre-specified measurable objectives, which would help to further define success. We have therefore tempered conclusions about outcomes and will incorporate explicit aims into future evaluation cycles. We plan to conduct focus groups and an end-of-year program survey after one full academic cycle. We continue to iteratively refine both Threads and Block 2.0 through targeted adjustments informed by implementation experience and real-time resident feedback, including efforts to optimize preceptor matching, manage scheduling complexity, and promote balanced exposure across disciplines.

In conclusion, in its inaugural year the Threads and Block 2.0 curricular redesign took important steps towards its objective of promoting individualized education, increased exposure to longitudinal ambulatory patient care and mentorship to provide career-aligned development for internal medicine residents. As ACGME continues to encourage competency-based and personalized training models, our approach offers a scalable and adaptable framework for residency programs seeking to foster professional identity formation while still meeting the core missions of internal medicine training.

## Data Availability

Data available from author upon request.
